# A phase II trial of bryostatin 1 in patients with non-Hodgkin's lymphoma

**DOI:** 10.1054/bjoc.2000.1624

**Published:** 2001-02

**Authors:** F H Blackhall, M Ranson, J A Radford, B W Hancock, M Soukop, A T McGown, A Robbins, G Halbert, G C Jayson for the Cancer Research Campaign Phase I/II Committee

**Affiliations:** CRC Department of Medical Oncology, Christie Hospital NHS Trust, Wilmslow Road, Withington, Manchester; YCRC Department of Clinical Oncology, Weston Park Hospital NHS Trust, Whitham Road, Sheffield; Department of Medical Oncology, Royal Infirmary, 84 Castle Street, Glasgow; CRC Drug Development Group, Paterson Institute for Cancer Research, Wilmslow Road, Withington, Manchester; Drug Development Office, Cancer Research Campaign, 10 Cambridge Terrace, London; CRC Formulation Unit, Department of Pharmaceutical Sciences, University of Strathclyde, Royal College Building, 204 George Street, Glasgow, UK

**Keywords:** bryostatin 1, non-Hodgkin's lymphoma, protein kinase C inhibitors

## Abstract

Bryostatin 1 is a naturally occurring macrocyclic lactone with promising antitumour and immunomodulatory function in preclinical and phase I clinical investigations. In this phase II study, 17 patients with progressive non-Hodgkin's lymphoma of indolent type (NHL), previously treated with chemotherapy, received a median of 6 (range 1–9) intravenous infusions of 25 μg/m^2^bryostatin 1 given once weekly over 24 hours. In 14 evaluable patients no responses were seen. Stable disease was attained in one patient for 9 months. The principal toxicities were myalgia and phlebitis. Treatment was discontinued early because of toxicity alone (phlebitis) in 2 patients, toxicity in addition to progressive disease in 3 patients (myalgia and phlebitis *n* = 2; thrombocytopenia *n* = 1) and progressive disease in 5 patients. The results fail to demonstrate efficacy of this regimen of bryostatin 1 in the treatment of NHL. In light of preclinical data that demonstrate synergy between bryostatin 1 and several cytotoxic agents and cytokines, clinical studies to investigate bryostatin 1 in combination are warranted. We also present data to demonstrate that central venous lines may be used in future studies to avoid phlebitis. © 2001 Cancer Research Campaign http://www.bjcancer.com


*British Journal of Cancer* (2001) **84**(4), 465–469
© 2001 Cancer Research Campaign
doi: 10.1054/ bjoc.2000.1624, available online at http://www.idealibrary.com on IDEAL®

### A phase II trial of bryostatin 1 in patients with non-Hodgkin's lymphoma

**FH Blackhall[1], M Ranson[1], JA Radford[1], BW Hancock[2], M Soukop[3], AT McGown[4], A Robbins[5], G Halbert[6] and GC Jayson[1] for the Cancer Research Campaign Phase I/II Committee**

[1]CRC Department of Medical Oncology, Christie Hospital NHS Trust, Wilmslow Road, Withington, Manchester; [2] YCRC Department of Clinical Oncology, Weston Park Hospital NHS Trust, Whitham Road, Sheffield; [3] Department of Medical Oncology, Royal Infirmary, 84 Castle Street, Glasgow; [4]CRC Drug Development Group, Paterson Institute for Cancer Research, Wilmslow Road, Withington, Manchester; [5] Drug Development Office, Cancer Research Campaign, 10 Cambridge Terrace, London; [6] CRC Formulation Unit, Department of Pharmaceutical Sciences, University of Strathclyde, Royal College Building, 204 George Street, Glasgow, UK

**Summary** Bryostatin 1 is a naturally occurring macrocyclic lactone with promising antitumour and immunomodulatory function in preclinical and phase I clinical investigations. In this phase II study, 17 patients with progressive non-Hodgkin's lymphoma of indolent type (NHL), previously treated with chemotherapy, received a median of 6 (range 1–9) intravenous infusions of 25 μg/m$^2$ bryostatin 1 given once weekly over 24 hours. In 14 evaluable patients no responses were seen. Stable disease was attained in one patient for 9 months. The principal toxicities were myalgia and phlebitis. Treatment was discontinued early because of toxicity alone (phlebitis) in 2 patients, toxicity in addition to progressive disease in 3 patients (myalgia and phlebitis $n = 2$; thrombocytopenia $n = 1$) and progressive disease in 5 patients. The results fail to demonstrate efficacy of this regimen of bryostatin 1 in the treatment of NHL. In light of preclinical data that demonstrate synergy between bryostatin 1 and several cytotoxic agents and cytokines, clinical studies to investigate bryostatin 1 in combination are warranted. We also present data to demonstrate that central venous lines may be used in future studies to avoid phlebitis. © 2001 Cancer Research Campaign http://www.bjcancer.com

**Keywords**: bryostatin 1; non-Hodgkin's lymphoma; protein kinase C inhibitors

Bryostatin 1 is a naturally occurring macrocyclic lactone derived from the marine invertebrate *Bugula neritina* (Pettit et al, 1982). It is a partial agonist of protein kinase C (PKC), a multigene family of isoenzymes with serine-threonine kinase activity that are crucial in cellular signalling pathways and influence proliferation and differentiation (Nishizuka, 1986). Bryostatin 1 induces differentiation of non-Hodgkin's lymphoma cell lines (Mohammed et al, 1993) and has antitumour activity against a variety of human and murine cell lines in vitro in addition to murine models of L10A B cell lymphoma in vivo (Pettit et al, 1982; Hornung et al, 1992). The exact mechanism of action of bryostatin 1 is unclear. It is known that an initial cellular effect is activation and translocation of PKC followed by its down regulation (Berkow et al, 1993). The antitumour effects of bryostatin 1 in vivo may in part be due to immunomodulatory function. For example, the expansion of myeloid and erythroid progenitor cells stimulated by the cytokines GM-CSF, M-CSF and IL-3 is amplified in the presence of bryostatin 1 (May et al, 1987; Sharkis et al, 1990). Similarly, peripheral blood mononuclear cells derived from cancer patients following intravenous infusion of bryostatin 1 have been shown to exhibit enhanced lymphokine activated killer cell activity and proliferation when stimulated by interleukin-2 (Scheid et al, 1994; Jayson et al, 1995). However bryostatin 1 also inhibits production of members of the matrix metalloproteinase family thought to be essential for angiogenesis and metastasis (Wojtowicz-Praga et al, 1997), down-regulates MDR1 gene expression (Al-Katib et al, 1998), modulates bcl-2 and p53 gene expression (Maki et al, 1995) and induces apoptosis (Mohammed et al, 1995) in models of human diffuse large cell lymphoma.

During phase I clinical evaluation of bryostatin 1 antitumour activity was observed in metastatic melanoma (Philip et al, 1993), ovarian cancer and low grade non-Hodgkin's lymphoma (Jayson et al, 1995). The dose-limiting toxicity (DLT) was myalgia and despite several investigations into the aetiology no effective antidote or treatment has been determined for this to date (Hickman et al, 1995; Thompson et al, 1996). Phlebitis was also a significant toxicity and initially attributed to the 60% ethanol formulation used for administration (Prendiville et al, 1993). The subsequent use of a PET formulation (10 μg bryostatin ml$^{-1}$ of 60% polyethylene glycol, 30% ethanol, 10% Tween 80) reduced the incidence of phlebitis (Philip et al, 1993). From these studies a maximum tolerated dose (MTD) of 25 μg/m$^2$ bryostatin 1 administered by infusion over one hour, weekly, for 3 weeks out of 4 (Philip et al, 1993), or over 24 hours once weekly (Jayson et al, 1995) was established. On the basis of the aforementioned preclinical and phase I data we undertook a phase II study to determine the efficacy of bryostatin 1 in patients with progressive NHL of indolent type.

*Received 29 June 2000*
*Revised 25 October 2000*
*Accepted 21 November 2000*

*Correspondence to:* G Jayson

### MATERIALS AND METHODS

### Patients

Patients eligible for inclusion were aged 18 or over with histologically proven NHL of indolent type, bi-dimensionally measurable and progressive disease. Patients could have received a maximum of two prior multi-drug chemotherapy regimens. Biopsy at relapse was recommended since the histological grade of NHL can change over time. Histological subtye was classified according to the updated Kiel Classification (Stansfeld et al, 1988). Patients were required to have a WHO performance status of 0–2, a life expectancy of greater than 3 months, a neutrophil count equal to or greater than $1.5 \times 10^9\ l^{-1}$, platelets equal to or greater than $100 \times 10^9\ l^{-1}$, serum transaminases less than $2.5 \times$ upper limit of normal, serum bilirubin less than or equal to 20 μM, serum creatinine less than or equal to 120 μM and no toxic manifestations of previous treatment except alopecia. Patients were excluded if they had severe or uncontrolled non-malignant systemic disease, active infection, previous or existing CNS disease, previous or concurrent malignancies except in situ carcinoma of the cervix or adequately treated basal or squamous cell carcinoma of the skin, if pregnant or lactating and if unable to give written informed consent. Concomitant treatment with systemic steroids was not permitted. The study was approved by the Phase I/II Committee and Central Institutional Review Board of the Cancer Research Campaign, the National Cancer Institute, Local Regional Ethics Committees and conducted according to the Declaration of Helsinki. Written informed consent was obtained in all patients. The use of bryostatin had UK Medicines Control Agency approval.

### Drug dose and administration

Bryostatin 1 (US National Cancer Institute, Arizona State University/ Cancer Research Institute, USA) was stored at 4˚C in vials containing 0.1 mg of lyophilized powder. For administration the lyophilized powder was dissolved in 1 ml of polyethylene glycol 400, ethanol and Tween 80 (PET, 60/30/10 v/v) then further diluted with 0.9% sodium chloride to give a solution containing 10 μg $ml^{-1}$ of bryostatin. This primary solution was further diluted by coinfusion with 1–2 litres of 0.9% saline over 24 hours through a peripheral venous catheter with the infusion rate of bryostatin controlled by a syringe pump. 10 ml polypropylene plastic syringes (SIMS Deltec Inc St Paul, MN, USA) and polyfin extension sets (model 126, Minimed Technologies, CA, USA) were used.

### Assessment of toxicity

Investigations performed before commencing therapy included a bone marrow trephine biopsy, full blood count with a differential white cell count, serum biochemistry, urinalysis and chest radiograph. Patients were reviewed by a physician weekly to record new signs and symptoms and document performance status (WHO). A full blood count with differential white cell count and serum biochemistry were repeated weekly. Additional investigations were performed as appropriate. National Cancer Institute of Canada Clinical Trials Group (NCIC-CTG) expanded common toxicity criteria were used to grade adverse events except for myalgia, which was graded according to the scale described by Philip et al (1993).

### Assessment of tumour response

Evaluable and measurable disease sites were assessed before treatment by physical examination, plain radiography and computerized tomography. Physical examination was repeated weekly and imaging investigations to determine tumour measurements were repeated monthly or at the time of suspected disease progression. Standard WHO criteria for assessment of objective responses were employed (Miller et al, 1981). Patients with progressive disease were withdrawn from the study. Patients were considered evaluable for response if they received 3 or more infusions of bryostatin.

### Statistics

To ensure a low probability ($P < 0.05$) of erroneously rejecting a treatment that is active in 20% of patients, a minimum of 14 evaluable patients were treated according to previously described principles (Gehan, 1961).

### Bryostatin adsorption studies

The extent of adsorption of bryostatin 1 onto the plastics used was examined. The materials examined were 10 ml polypropylene syringe (SIMS Deltec Inc, St Paul, MN, USA), polyfin extensions sets (MiniMed Technologies, Sylmar, CA, USA) and central venous catheter (Broviac 6.6 Fr single lumen, Bard Ltd, Crawley, UK). Bryostatin 1 solutions were prepared exactly as for clinical drug administration at 10/μg ml and a typical 40 μg dose of bryostatin 1 was used to fill the infusion devices. Following storage at room temperature in standard lighting samples were withdrawn and analysed by UV-HPLC at time points up to 7 days after filling according to previously published methodology (Khan et al, 1998). Concentrations of bryostatin 1 were determined by use of standard curves run immediately before the samples. All plastics were tested in duplicate and duplicate drawn samples from each set were analysed.

### RESULTS

### Patients

17 patients (10 men, 7 women: age range 39–77 years, median 56, mean 58) with NHL were recruited. 16 patients had previously received chemotherapy including an alkylating agent (≤ 2 single drug regimens; $n$ = 6: ≤2 multidrug regimens ± ≥1 single drug regimen; $n$ = 9: >2 multidrug regimens; $n$ = 1). 7 patients had also received prior radiotherapy, 3 patients had also received biological therapy (vitamin D and/or interferon) and 1 patient had received PUVA therapy. Re-biopsy evidence of low-grade NHL was obtained in 14 patients and all had documented disease progression within 2 months prior to entry to the study. Their characteristics are summarized in Table 1.

### Response to treatment

Of 17 patients treated, 14 were evaluable for response. Of those who were not evaluable, 2 patients received less than 3 infusions and 1 patient had received more than 2 previous multidrug regimens. The median number of bryostatin 1 infusions given per patient was 6 (range 1–9) and 7 patients received 8 or more infusions. The outcomes of treatment are summarized in Table 2. No responses (complete or partial) were seen although there were mixed responses in 6 patients with some lesions undergoing shrinkage and others progressing. In one patient disease stabilization was for 9 months. This patient declined further treatment after 8 infusions in order to return to work.

© 2001 Cancer Research Campaign

**Table 1** Patient characteristics ($n$ = 17)

| Parameter | No. of patients |
|---|---|
| Sex | |
| F | 7 |
| M | 10 |
| Age | median = 56 years (range 39–77) |
| WHO performance status | |
| 0 | 11 |
| 1 | 5 |
| 2 | 1 |
| 3 | 0 |
| 4 | 0 |
| Disease sites | |
| Nodal | 13 |
| Nodal and skin | 1 |
| Nodal and pleural | 1 |
| Nodal and liver | 1 |
| Skin | 1 |
| Bone marrow involvement | 13 |
| Histology | |
| Follicular | 7 |
| Small lymphocytic | 4 |
| Other indolent B cell types | 6 |
| Previous chemotherapy | |
| Alkylating agent | 16 |
| Anthracycline | 4 |

### Toxicity

All patients were included in the analysis of toxicity (Table 3). The main toxicities were myalgia ($n$ = 8) and phlebitis ($n$ = 13). A one week treatment delay and dose reduction (25%) of bryostatin 1 in one patient who had grade 3 myalgia prevented subsequent episodes. The median number of bryostatin infusions given prior to onset of myalgia and phlebitis was 2 (range 1–9) and 1 (range 1–4), respectively. Treatment was withdrawn in 4 patients due to phlebitis. In one patient bryostatin 1 was discontinued after 5 infusions due to grade 2 thrombocytopenia which was possibly treatment related.

### Bryostatin adsorption studies

Bryostatin 1 was assayed by our previously published method (Khan et al, 1998). No visible colour changes or precipitate formed upon storage for up to 7 days. There were no additional or apparent decomposition peaks as assessed by HPLC profiles. Adsorption to the polypropylene infusion device, extension set and central venous catheter was very low at 24 hours and upon storage for 7 days there was greater but limited adsorption to the infusion device (Figure 1). This adsorption data is similar to that reported by others (Cheung et al, 1998). It should be noted that PVC shows significant adsorptive properties (Cheung et al, 1998) and our own preliminary work suggests that ethyl vinyl acetate also adsorbs bryostatin 1 (AT McGown, M Ranson, unpublished observations).

### DISCUSSION

In this phase II study 17 patients with progressive NHL, previously treated with chemotherapy, received a median of 6 (range 1–9) intravenous 24 hour infusions of 25 μg/m$^2$ bryostatin 1, given once weekly. 7 patients completed 8 or more infusions. No responses were observed although stable disease was attained in one patient for 9 months. The majority (11/17) of patients were withdrawn from the study because of disease progression and in 5 patients this occurred before 8 infusions of bryostatin 1 had been administered.

The reason for lack of efficacy despite promising preclinical and phase I data, is unclear. Phase II studies of bryostatin 1 given at the same dose but with a one hour infusion in patients with malignant melanoma have also failed to demonstrate significant antitumour activity (Propper et al, 1998; Gonzalez et al, 1999). In contrast, in a phase I trial Varterasian et al (1998) achieved a higher MTD, again limited by myalgia, of 120 μg/m$^2$ bryostatin 1, infused over 72 hours every 2 weeks. A phase II trial of this regimen was recently reported documenting one complete remission of 18 months and two partial remissions of greater than 6 months duration in patients with low grade NHL (Varterasian et al, 2000). Lack of efficacy of bryostatin 1 in the current study may therefore be due to suboptimal dose and duration of treatment but antitumour

**Table 2** Outcome of treatment with bryostatin ($n$=17)

| Patient | Number of infusions (weeks on treatment) | Reason off study | Response |
|---|---|---|---|
| 1 | 5 (6**) | PD | PD |
| 2 | 6 (6) | Toxicity (phlebitis + myalgia) + PD | PD* |
| 3 | 4 (4) | Toxicity (phlebitis + myalgia) + PD | PD |
| 4 | 6 (7**) | PD | PD |
| 5 | 8 (8) | PD | PD |
| 6 | 5 (5) | Toxicity (thrombocytopenia) + PD | PD* |
| 7 | 2 (2) | PD | Not evaluable |
| 8 | 8 (8) | PD | PD |
| 9 | 9 (10**) | PD | PD |
| 10 | 3 (3) | PD | PD |
| 11 | 8 (8) | PD | PD |
| 12 | 1 (1) | Toxicity (phlebitis) | Not evaluable |
| 13 | 5 (5) | Toxicity (phlebitis) | Not evaluable |
| 14 | 9 (11**) | PD | PD |
| 15 | 8 (8) | Declined further treatment | Stable disease |
| 16 | 8 (9**) | PD | PD |
| 17 | 3 (4**) | PD | PD |

*Clinical evidence of disease progression, objective measurements were not evaluable. **Patients in whom treatment delays occurred. PD = progressive disease.

*© 2001 Cancer Research Campaign*

**Table 3** Toxicities associated with bryostatin treatment ($n$ = 17)

| | Number of patients | | | | |
|---|---|---|---|---|---|
| **NCIC – CTG Grade*** | **0** | **1** | **2** | **3** | **4** |
| Myalgia** | 9 | 4 | 3 | 1 | 0 |
| Phlebitis | 4 | 1 | 10 | 2 | 0 |
| Headache | 14 | 3 | 0 | 0 | 0 |
| Fatigue | 12 | 5 | 0 | 0 | 0 |
| Nausea/vomiting | 13 | 4 | 0 | 0 | 0 |
| Diarrhoea | 16 | 1 | 0 | 0 | 0 |
| Thrombocytopenia | 15 | 1 | 1 | 0 | 0 |
| Leucopenia | 15 | 2 | 0 | 0 | 0 |
| Bilirubin | 16 | 0 | 0 | 1 | 0 |
| Neuralgia | 16 | 1 | 0 | 0 | 0 |

*National Cancer Institute of Canada Clinical Trials Group expanded toxicity scale. **Graded according to Philip et al (1993).

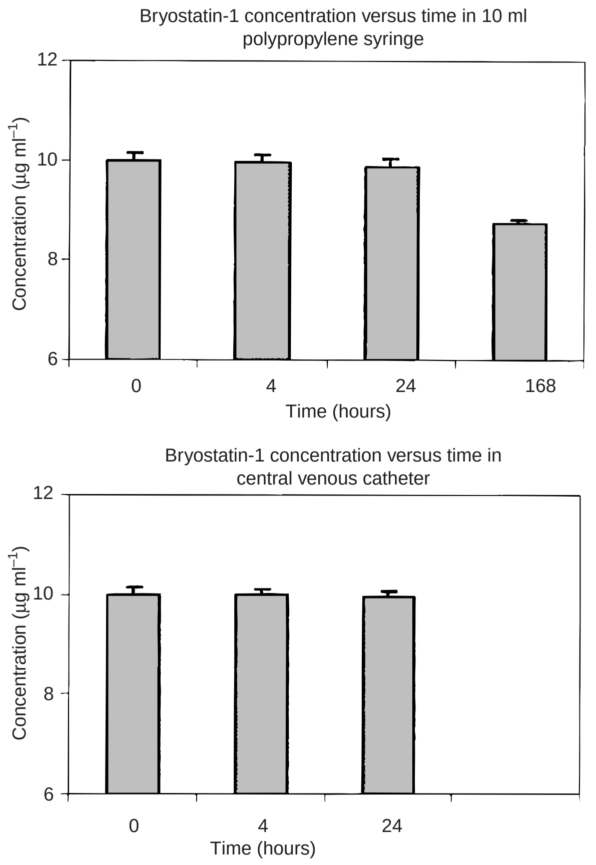

**Figure 1** Adsorption of bryostatin 1 onto infusion devices activity was observed during phase I evaluation using both 1 and 24 hour infusions of 25 μg/m$^2$ bryostatin 1 (Philips et al, 1993; Jayson et al, 1995). As there is no established method to reliably determine serum concentrations of bryostatin 1 in humans it has not been possible to obtain pharmacokinetic data to determine serum concentrations and rationally optimize the schedule. Animal data suggest that bryostatin 1 has a short plasma half life (Berkow et al, 1993) with in vitro and in vivo data showing enhanced antitumour effects on prolonged exposure (Hornung et al, 1992) and the data of Varterasian et al would support this. However, it is perplexing that significant differences in MTD of bryostatin 1 have been demonstrated despite consensus regarding toxicity. In addition to the aforementioned studies a MTD of 44 μg/m$^2$ bryostatin 1 administered over 1 hour weekly for 3 weeks out of 4 has been reported in a paediatric oncology group study (Weitman et al, 1999). The significant adsorption of bryostatin 1 onto polyvinyl chloride and ethyl vinyl acetate (Cheung et al, 1998) raises the possibility that differences in adsorptive properties of administration devices used may account for discrepancies in MTD observed. Compared with other studies, phlebitis was a significant toxicity in this study. We chose a peripheral vein for drug administration due to uncertainty over the adsorption of bryostatin onto material used for central infusion devices but have subsequently demonstrated that the adsorption of bryostatin onto small (10 ml) polypropylene infusion devices and a central infusion catheter is negligible over 24 hours (Figure 1). Central administration may therefore be safely used to avoid phlebitis; however materials used for infusion of bryostatin 1 should be clearly stated in all reports of clinical trials.

Further explanations for lack of efficacy of bryostatin 1 in this study include suppressed lymphocyte function due to lymphoma or previous chemotherapy and radiotherapy which may have prevented bryostatin 1 from acting through immune stimulatory mechanisms (Propper et al, 1998). In addition the modulation of tumour-specific PKC isoenzyme profiles by bryostatin 1 is poorly understood. PKC isoenzymes are involved in both oncogene and tumour suppressor gene activation, variable expression of PKC isotypes in tumours has been demonstrated and the degree to which isotypes are downregulated by bryostatin 1 also varies (Buchner, 2000). Bryostatin 1 may only be effective when targeted to individuals bearing tumours with particular PKC isoenzyme profiles.

The efficacy of bryostatin 1 may be enhanced by administration in combination. For example, pretreatment with bryostatin 1 increases the cytotoxicity of 2-chlorodeoxyadenosine in drug-resistant chronic lymphocytic leukaemia cells (Mohammed et al, 1998), cisplatin in human cervical carcinoma cells (Basu and Lazo, 1992) and cytarabine in fresh blast cells from patients with acute myeloid leukaemia (Elgie et al, 1998). In a tumour-bearing mouse model enhanced cytotoxicity is observed when bryostatin 1 is administered following paclitaxel (Koutcher et al, 2000); synergy between bryostatin 1 and tamoxifen, which also inhibits PKC, has been demonstrated in the drug resistant P388 leukaemia cell line which lacks steroid receptors (McGown et al, 1998) and vincristine in combination with bryostatin 1 has been shown to cure mice bearing xenografts of neoplastic B cells derived from human Waldenströms macroglobulinaemia (Mohammed et al, 1994). On this basis, Varterasian et al (2000) conducted a feasibility study in which patients who developed progressive NHL while receiving single agent bryostatin were given sequential treatment with vincristine. Doses of up to 2 mg/m$^2$ vincristine were well tolerated with no unexpected or enhanced toxicity. Similarly, in early reports of phase I trials, bryostatin 1 in combination with paclitaxel or cisplatin appears to be well tolerated and myalgia has occured less frequently than in single agent trials (Kaubisch et al, 1999; Rosenthal et al, 1999).

In summary, this study failed to show a significant benefit from single agent bryostatin 1 in progressive NHL of indolent type. Improved understanding of bryostatin 1 pharmacokinetics and

 © 2001 Cancer Research Campaign modulation of tumour PKC isotypes by bryostatin 1 would probably aid development of this novel agent. Further evaluation of bryostatin 1 in combination is warranted.

### ACKNOWLEDGEMENTS

The authors would like to thank G Connoly, Data Manager, J Clayton and A Watson, Research Nurses, at The Christie Hospital; V Evans, Clinical Nurse Specialist, at Glasgow Royal Infirmary; Sarah Armstrong and the Drug Development Office at the CRC for their trial management and the NCI for provision of bryostatin 1. Fiona Blackhall is supported by a CRC Research Fellowship for a Clinician.

### REFERENCES

Al-Katib AM, Smith MR, Kamanda WS, Pettit GR, Hamdan M, Mohamed AN, Chelladurai B and Mohammed RM (1998) Bryostatin 1 down-regulates mdr1 and potentiates vincristine cytotoxicity in diffuse large cell lymphoma xenografts. *Clin Cancer Res* **4**: 1305–1314

Basu A and Lazo JS (1992) Sensitisation of human cervical carcinoma cells to cis-diamminediachloroplatinum(II) by bryostatin 1. *Cancer Res* **52**(11): 3119–3124

Berkow RL, Schlabach L, Dodson R, Benjamin WJ, Pettit GR, Rustagi P and Kraft AS (1993) In vivo administration of the anticancer agent bryostatin 1 activates platelets and neutrophils and modulates protein kinase C activity. *Cancer Res* **53**: 2810–2815

Buchner K (2000) The role of protein kinase C in the regulation of cell growth and in signalling to the cell nucleus. *J Cancer Res Clin Oncol* **126**(1): 1–11

Cheung AP, Hallock YF, Vishnuvajjala BR, Nguyenle T and Wang E (1998) *Invest New Drugs* **16**(3): 227–236

Elgie AW, Sargent JM, Alton P, Peters GJ, Noordhuis P, Williamson CJ and Taylor CG (1998) Modulation of resistance to ara-C by bryostatin in fresh blast cells from patients with AML. *Leuk Res* **22**(4): 373–378

Gehan A (1961) The determination of the number of patients required in a preliminary and a follow up trial of a new chemotherapeutic agent. *J Chronic Dis* **13**: 346–353

Gonzalez R, Ebbinghaus S, Henthorn TK, Miller D and Kraft AS (1999) Treatment of patients with metastatic melanoma with bryostatin 1 – a phase II study. *Melanoma Res* **9**(6): 599–606

Hickman PF, Kemp GJ, Thompson CH, Salisbury AJ, Wade K, Harris AL and Radda GK (1995) Bryostatin 1, a novel antineoplastic agent and protein kinase C activator, induces human myalgia and muscle metabolic defects: a 31P magnetic resonance spectroscopic study. *Br J Cancer* **72**(4): 998–1003

Hornung RL, Pearson JW, Beckwith M and Longo DL (1992) Preclinical evaluation of bryostatin as an anticancer agent against several murine tumour cell lines: in vitro versus in vivo activity. *Cancer Res* **52**: 101–107

Jayson GC, Crowther D, Prendiville J, McGown A, Scheid C, Stern P, Young R, Brenchley P, Chang J and Owens S and Pettit GR (1995) A phase I trial of bryostatin 1 in patients with advanced malignancy using a 24 hour intravenous infusion. *B J Cancer* **72**: 461–468

Kaubisch A, Kelsen DP, Saltz L, Kemeny N, O'Reilly E, Ilson D, Endres S, Barazzuol J and Schwartz GK (1999) A phase I trial of weekly sequential bryostatin-1 and paclitaxel in patients with advanced solid tumours. *Proceedings of the American Society of Clinical Oncology* Abstract 639

Khan P, McGown AT, Dawson MJ, Jayson G, Prendiville JA, Pettit GR and Crowther D (1998) High Performance liquid chromatographic assay for the novel antitumour drug, bryostatin-1, incorporating a serum extraction technique. *J Chromatogr B Biomed Sci Appl* **709**(1): 113–117

Koutcher JA, Motwani M, Zakian KL, Li XK, Matei C, Dyke JP, Ballon D, Yoo HH and Schwartz GK (2000) The in vivo effect of bryostatin 1 on paclitaxel-induced tumour growth mitotic entry, and blood flow. *Clin Cancer Res* **6**(4): 1498–1507

Maki A, Diwakaran H, Redman B, Al-Asfar S, Pettit GR, Mohammed RM and Al-Katib A (1995) The bcl-2 and p53 oncoproteins can be modulated by bryostatin 1 and dolastatins in human diffuse large cell lymphoma. *Anticancer Drugs* **6**: 392–397

May WS, Sharkis SJ, Esa AH, Gebbia U, Kraft AS, Pettit GR and Sensenbrenner L (1987) Antineoplastic bryostatins are multipotential stimulators of human haematopoietic progenitor cells. *Proc Natl Acad Sci* **84**: 8483–8487

McGown AT, Jayson G, Pettit GR, Haran MS, Ward TH and Crowther D (1998) Bryostatin 1 – tamoxifen combinations show synergistic effects on the inhibition of growth of P388 cells in vitro. *Br J Cancer* **77**(2): 216–220

Miller AB, Hoogstraten B, Staquet M and Winkler A (1981) Reporting results of cancer treatment. *Cancer* **47**(1): 207–214

Mohammed RM, Al-Katib A, Pettit RG and Sensenbrenner LL (1993) Differential effects of bryostatin 1 on human non-Hodgkin's lymphoma cell lines. *Leukaemia Research* **17**: 1–8

Mohammed RM, Al-Katib, Pettit GR and Sensenbrenner LL (1994) Successful treatment of human Waldenstrom's macroglobulinaemia with combination biological and chemotherapy agents. *Cancer Res* **54**: 165

Mohammed RM, Diwakaran H, Maki A, Emara MA, Pettit GR, Redman B and Al-katib A (1995) Bryostatin 1 induces apoptosis and augments inhibitory effects of vincristine in human diffuse large cell lymphoma. *Leuk Res* **19**: 667–673

Mohammed RM, Beck FW, Katato K, Hardy N, Wall N and Al-Katib A (1998) Potentiation of 2-chlorodeoxyadenosine activity by bryostatin 1 in the resistant chronic lymphocytic leukaemia cell line (WSU-CLL): association with increased ratios of dCK/5-NT and Bax/Bcl-2. *Biol Chem* **379**(10): 1253–1261

Nishizuka Y (1986) Studies and perspectives of protein kinase C. *Science* **233**: 305–312

Pettit GR, Herald CL and Doubek DL (1982) Isolation and structure of bryostatin 1. *J Am Chem soc* **104**: 6846–6848

Philip AP, Rea D, Thavasu P, Carmichael J, Stuart NSA, Rockett H, Talbot DC, Ganeson T, Pettit GR, Balkwill F and Harris AL (1993) Phase I study of bryostatin 1: Assessment of interleukin 6 and tumor necrosis factor α induction in vivo. *J Natl Cancer Institute* **85**(22): 1812–1818

Prendiville J, Crowther D, Thatcher N, Woll PJ, Fox BW, McGown A, Testa N, Stern P, McDermott R, Potter M and Pettit GR (1993) A Phase I study of intravenous bryostatin 1 in patients with advanced cancer. *Br J Cancer* **68**: 418–424

Propper DJ, Macaulay V, O'Byrne KJ, Braybrooke JP, Wilner SM, Ganesean TS, Talbot DC and Harris AL (1998) A phase II study of bryostatin 1 in metastatic malignant melanoma. *Br J Cancer* **78**(10): 1337–1341

Rosenthal MA, Oratz R, Liebes L, Cahr MH and Muggia FM (1999) Phase I study of bryostatin-1 (NSC 339555) and Cisplatin in Advanced Malignancies. *Proceedings of the American Society of Clinical Oncology* Abstract 873

Scheid C, Prendiville J, Jayson G, Crowther D, Fox B, Pettit GR and Stern PL (1994) Immunomodulation in patients receiving intravenous bryostatin 1 in a phase 1 clinical study: comparison with effects of bryostatin 1 on lymphocyte function in vitro. *Cancer Immunol Immunother* **39**: 223–230

Sharkis SJ, Jones RJ, Bellis ML, Demitri GD, Griffin JD, Civin C and May WS (1990) The action of bryostatin on normal human haematopoietic progenitors is mediated by accessory cell release of growth factors. *Blood* **76**: 716–720

Stansfeld AG, Diebold G, Noel H, Kapanci Y, Rilke F, Kelenyi G, Sundstrom C, Lennert K, van-Unnik JA, Mioduszewska O, et al (1988) Updated Kiel Classification for lymphomas (letter). *Lancet* **1**(8580): 292–293

Thompson CH, Macaulay VM, O'Byrne KJ, Kemp GJ, Wilner SM, Talbot DC, Harris AL and Radda GK (1996) Modulation of bryostatin 1 muscle toxicity by nifedipine: effects on muscle metabolism and oxygen supply. *Br J Cancer* **73**(10): 1161–1165

Varterasian ML, Mohammed RM, Eilender DS, Hulbard K, Rodriguez DH, Pemberton PA, Pluda JM, Dan MD, Pettit GR, Chen BDM and Al-Katib AM (1998) Phase I study of bryostatin 1 in patients with relapsed non-Hodgkin's lymphoma and chronic lymphocytic leukaemia. *J Clin Oncol* **16**(1): 56–62

Varterasian ML, Mohammed RM, Shurafa MS, Hulbard K, Pemberton PA, Rodriguez DH, Spadoni V, Eilender DS, Murgo A, Wall N, Dan M and Al-Katib AM (2000) Phase II trial of bryostatin 1 in patients with relapsed low-grade non-Hodgkin's lymphoma and chronic lymphocytic leukaemia. *Clin Cancer Res* **6**: 825–828

Weitman S, Langevin AM, Berkow RL, Thomas PJ, Hurwitz CA, Kraft AS, Dubowy RL, Smith DL and Bernstein M (1999) A Phase I trial of bryostatin 1 in children with refractory solid tumours: a Pediatric Oncology Group study. *Clin Cancer Res* **5**(9): 2344–2348

Wojtowicz-Praga SM, Dickson RB and Hawkins MJ (1997). Matrix metalloproteinase inhibitors. *Investigational New Drugs* **15**: 61–75

© 2001 Cancer Research Campaign 
